# A Rare Embryonic Arterial Anomaly: Thrombosis of Persistent Sciatic Artery

**DOI:** 10.7759/cureus.55023

**Published:** 2024-02-27

**Authors:** Akshaya K Panda, Sudhir Rai, Sandipan Mukhopadhyay, Zaid M Nafe

**Affiliations:** 1 Surgery, Tata Main Hospital, Jamshedpur, IND; 2 Radiology, Tata Main Hospital, Jamshedpur, IND; 3 Gastroenterology, Tata Main Hospital, Jamshedpur, IND

**Keywords:** dry gangrene, below-knee amputation, congenital vascular anomaly, lower limb ischemia, persistent sciatic artery (psa)

## Abstract

Persistent sciatic artery (PSA) is an exceptionally rare congenital vascular anomaly with profound clinical implications. This condition occurs when the primitive sciatic artery, responsible for fetal lower limb blood supply, fails to regress during embryonic development. PSA persists into adulthood, representing an intriguing vascular variation that can present as gluteal aneurism and thrombosis. We present the case of a 72-year-old female patient admitted with abdominal pain and blackening of her right foot. Clinical examination revealed dry gangrene affecting the toes, limb edema, and absent peripheral pulses in the right lower limb. Septic shock and electrolyte imbalances prompted immediate resuscitation and antibiotic therapy. Diagnostic investigations, including Doppler ultrasonography, CT angiography, and 2D echocardiography, identified a right-sided PSA. With limb ischemia being irreversible, a below-knee amputation was performed. This case highlights the clinical presentation, diagnostic workup, and management of a rare PSA, emphasizing the importance of prompt recognition and intervention in complex vascular anomalies.

## Introduction

Persistent sciatic artery (PSA) represents an intriguing and exceedingly rare vascular anomaly with profound clinical implications [1]. This congenital condition arises during embryonic development when the primitive sciatic artery, responsible for supplying the lower limb during fetal life, regresses as the fetal circulatory system matures [2]. However, in exceptional cases, this regression fails to occur, resulting in the persistence of the sciatic artery into adulthood [2]. This remarkable vascular variation can give rise to a spectrum of clinical challenges, making it a subject of great interest and significance in the field of vascular medicine [2].

PSA is an uncommon anatomical variation that was initially documented in 1832, and its angiographic characteristics were first outlined in 1960 [3]. It is an exceedingly rare vascular anomaly that stems from the failure of a fetal arterial structure to regress during embryonic development. This condition, with a prevalence estimated at approximately 0.02%-0.05% of the population, represents an intriguing and infrequent occurrence in the field of vascular medicine. PSA's uniqueness lies in its distinct embryological origin, anatomical course, and clinical implications [4].

PSA is characterized by several noteworthy anatomical attributes as follows:

Origin: It typically originates from the internal iliac artery, a key branch of the common iliac artery, which is a major conduit in the pelvic vascular network.

Course: PSA closely parallels the sciatic nerve, running alongside it as it extends down the posterior aspect of the thigh to the knee.

Supply: One of the most significant features of PSA is its role as a major supplier to the popliteal artery, a vital conduit for blood flow to the lower leg and foot [5].

PSA can introduce a spectrum of clinical challenges and complications. These may include the following:

Lower limb ischemia: PSA can impede or disrupt blood flow to the lower extremities, potentially leading to lower limb ischemia.

Aneurysm formation: The abnormal course of PSA can make it susceptible to aneurysm formation in the gluteal region, further complicating the vascular landscape.

Hemorrhage risk: In certain cases, PSA aneurysm can be vulnerable to rupture, posing a risk of severe hemorrhage [6]. PSA typically presents with a range of clinical manifestations, although it can remain asymptomatic in some individuals. Symptoms may include intermittent claudication, pain in the affected limb, or even life-threatening hemorrhage in severe cases [7].

In this study, we describe the case of a patient with a PSA who presented with acute limb ischemia.

## Case presentation

**Figure 1:**
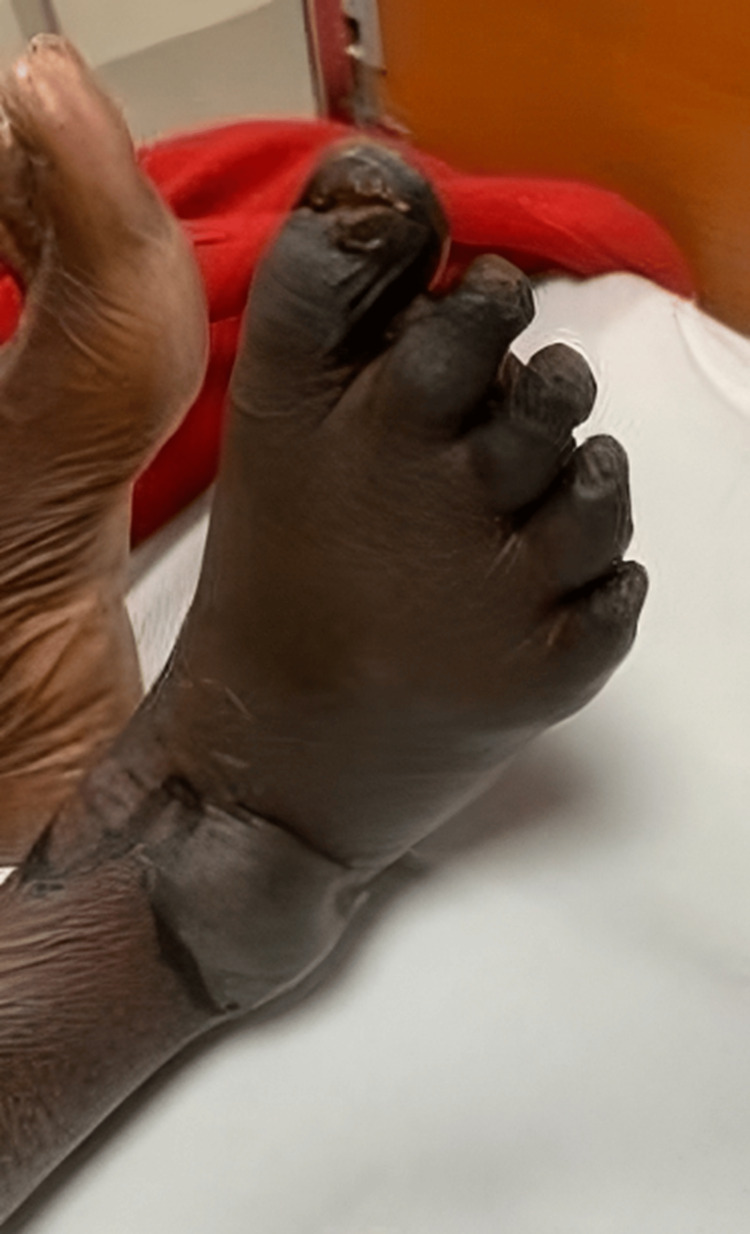
Dry gangrene was observed in all the toes of the right foot, and blisters were noted on the dorsum of the foot with a line of demarcation.

**Figure 2:**
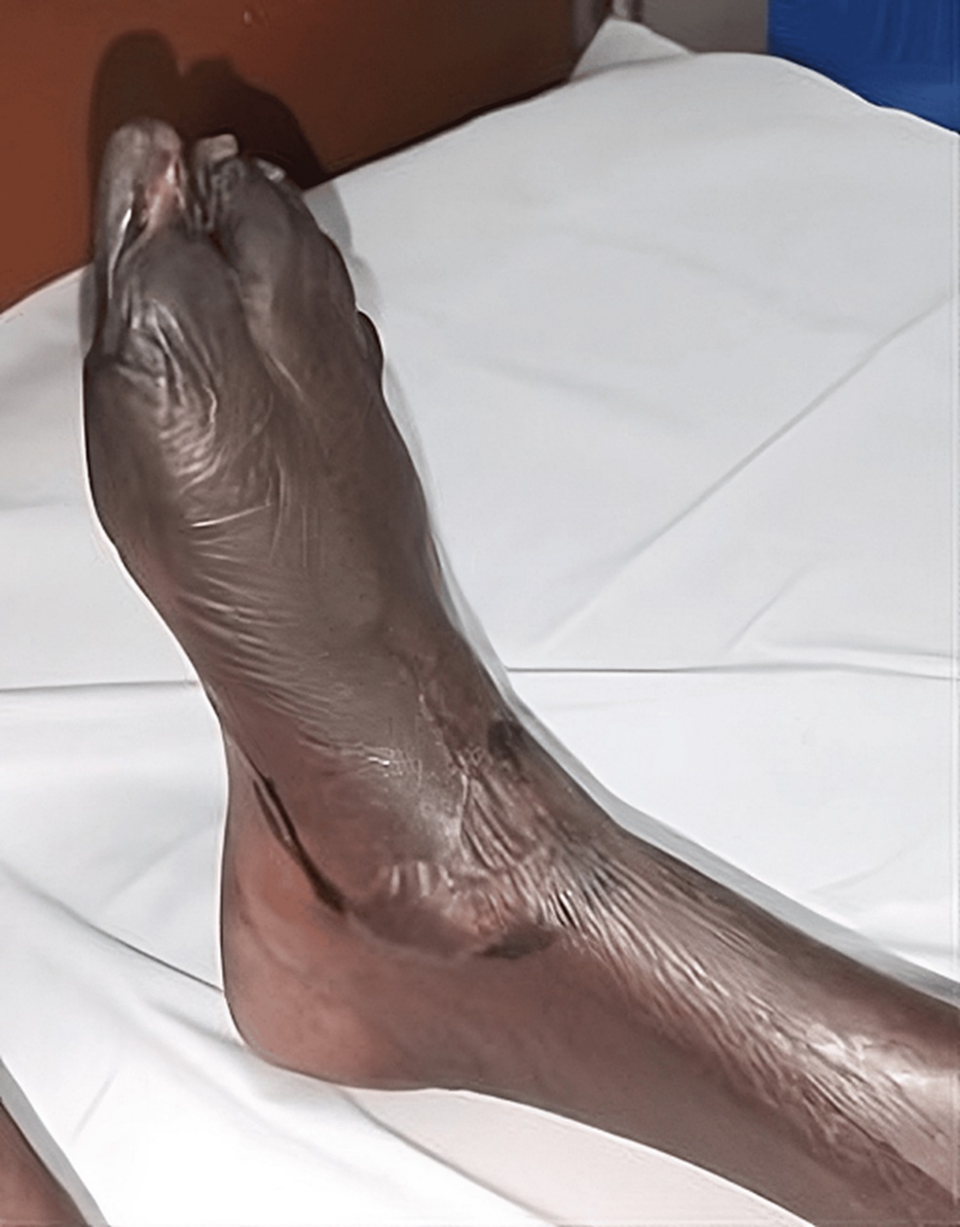
Dry gangrene was observed in all the toes of the right foot, and blisters were noted on the dorsum of the foot with a line of demarcation.

A 72-year-old female patient was admitted to our medical facility with the chief complaints of severe abdominal pain and an alarming symptom of blackening in her right foot, which had persisted for the past seven days. Her medical history included prior treatment for tuberculosis (TB) in 2018, and she had a notable history of tobacco use, spanning two to three decades. Upon admission, the patient’s clinical examination revealed several concerning findings:

Right foot: Dry gangrene was observed in all the toes of the right foot, with edema extending up to the knee. Blisters were noted on the dorsum of the foot with a line of demarcation (Figures 1-2).

Right lower limb: Below the knee, the right lower limb exhibited coldness and a clammy texture.

Peripheral pulses: Examination of peripheral pulses in the right lower limb revealed an absence of pulsation in the right popliteal artery, anterior tibial artery, and posterior artery. Femoral pulses were feebly palpable. The left lower limb pulses including the left femoral pulses were well felt.

Recognizing the patient's critical condition, characterized by septicemic shock and severe electrolyte imbalances, immediate resuscitation measures were initiated. Antibiotic therapy was promptly initiated because of an elevated total leukocyte count (TLC). To further assess and diagnose the underlying vascular issue, a series of diagnostic investigations were carried out:

Doppler ultrasonography (USG) of both limbs: The results indicated the occlusion of the right popliteal artery, accompanied by a complete absence of blood flow in the anterior and posterior tibial arteries. Following consultation with a physician, the patient was prescribed 60 mg enoxaparin injection twice daily as part of her treatment regimen.

2D echocardiogram (2D echo): This diagnostic test returned normal results, eliminating any cardiac abnormalities as contributing factors to her condition.

CT angiography of both lower limbs: The imaging findings from this diagnostic modality revealed severe narrowing and irregularities in the right superficial femoral artery, complete occlusion at the level of the right popliteal artery, and an absence of blood flow in the right dorsalis pedis artery.

**Figure 3:**
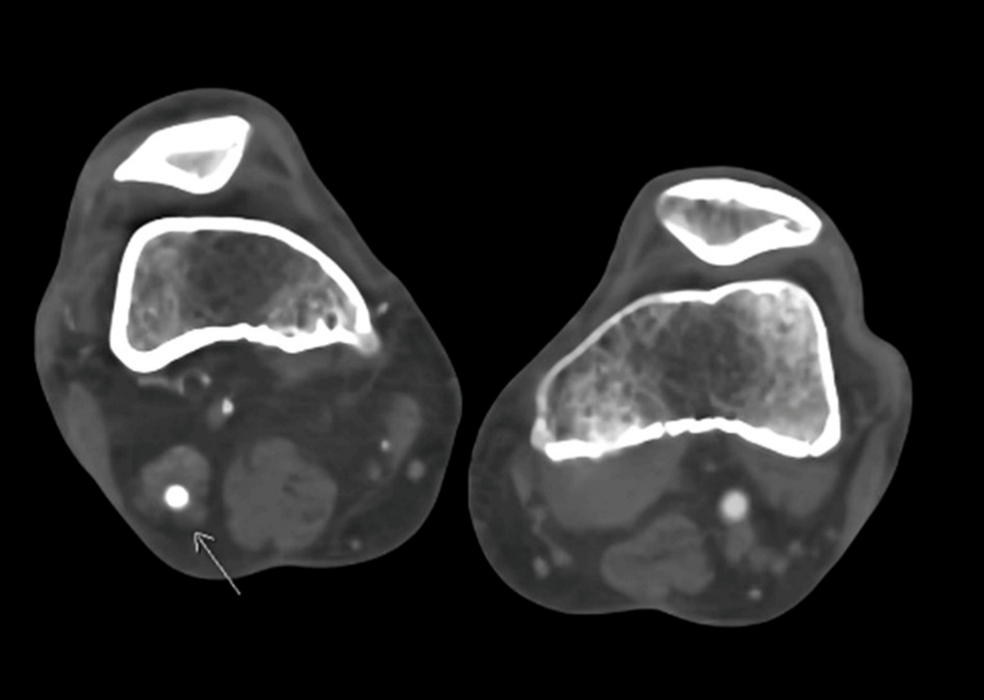
Right-sided PSA with patent lumen: axial source image.

**Figure 4:**
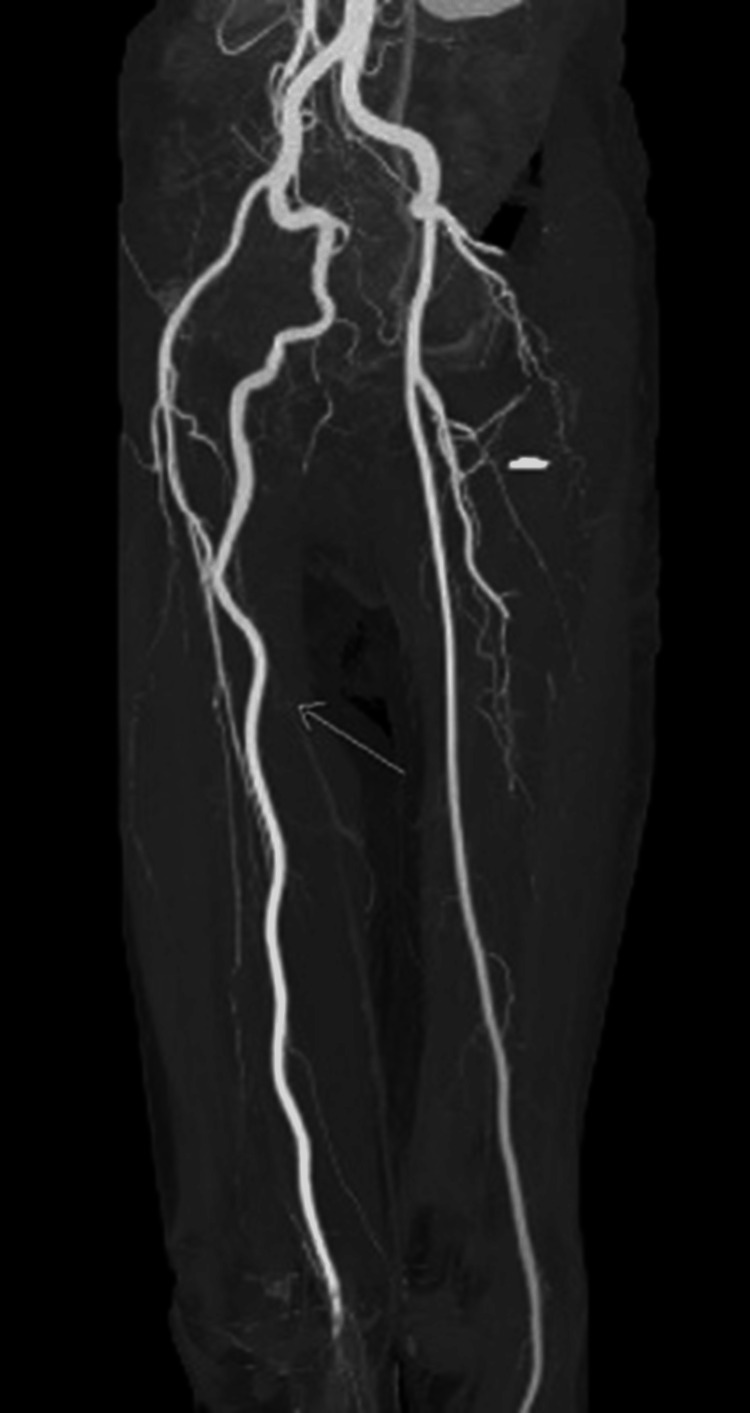
Right-sided PSA with patent lumen: multiplanar reformatted (MPR).

**Figure 5:**
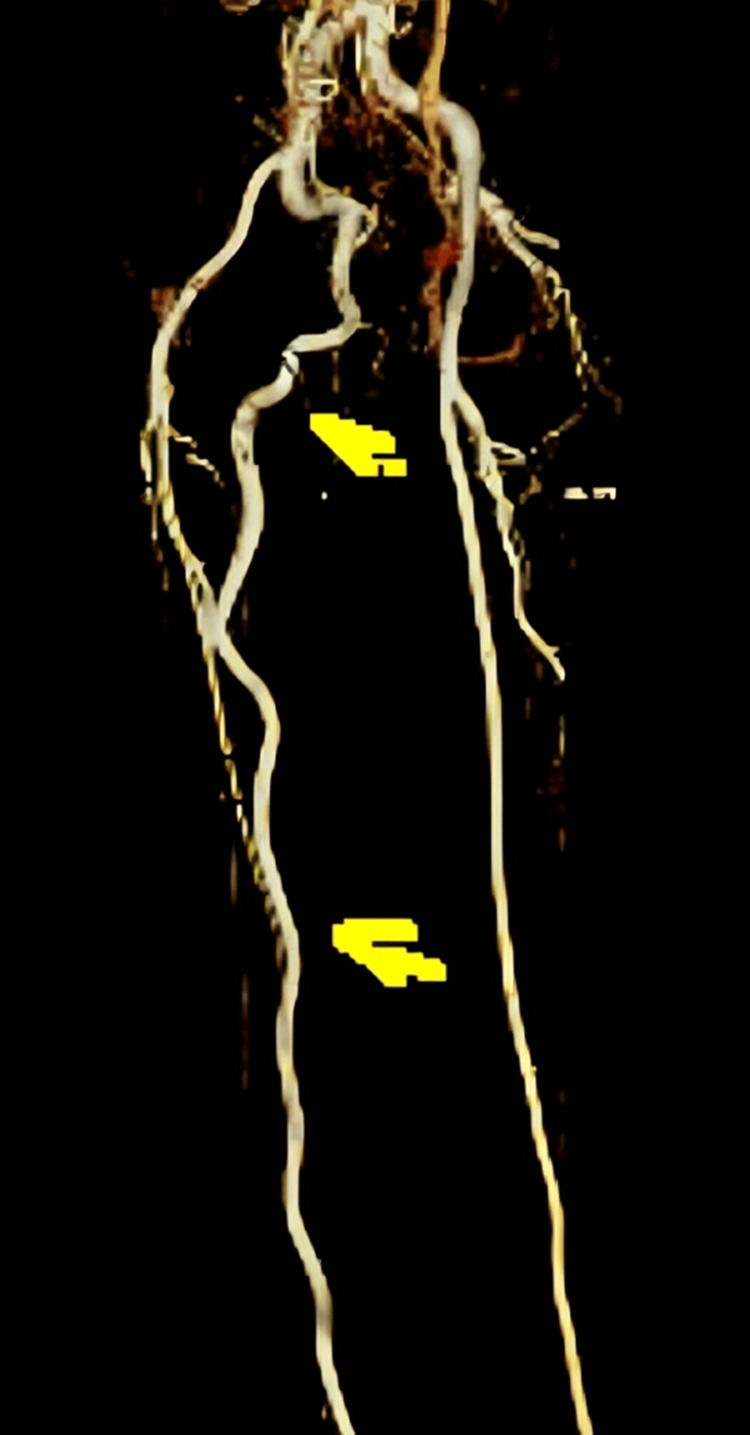
Right-sided PSA with patent lumen: volume rendering (VR) image.

**Figure 6:**
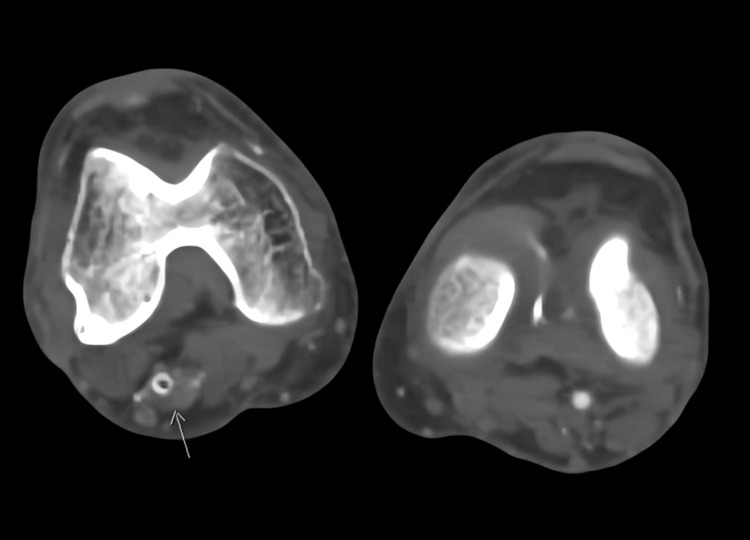
Axial section image demonstrates intraluminal filling defect (long arrow) indicating thrombus within the artery.

**Figure 7:**
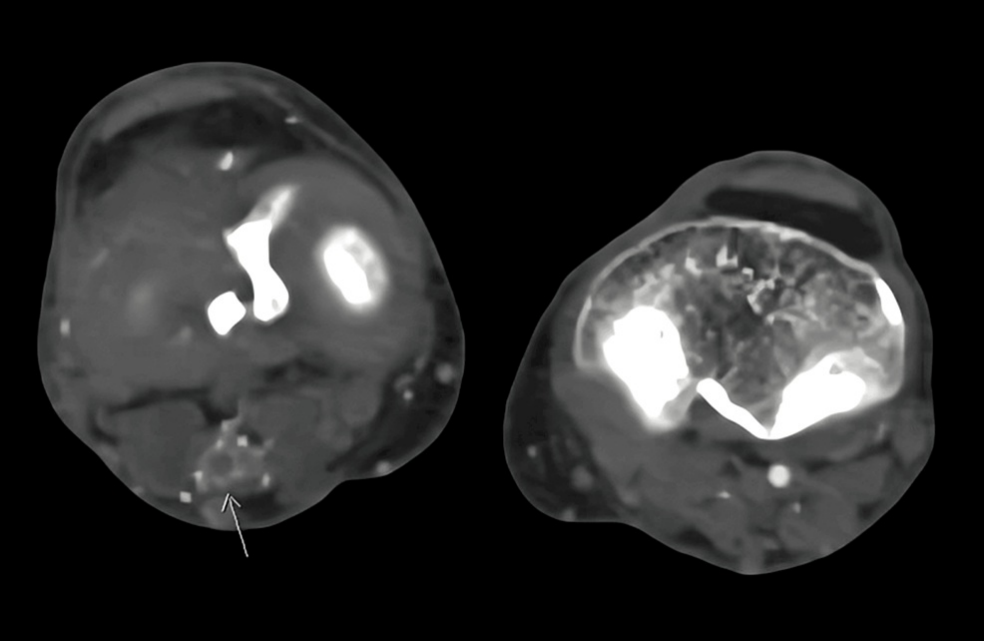
Axial slice pointed (arrows in images) to the level of complete occlusion of PSA.

**Figure 8:**
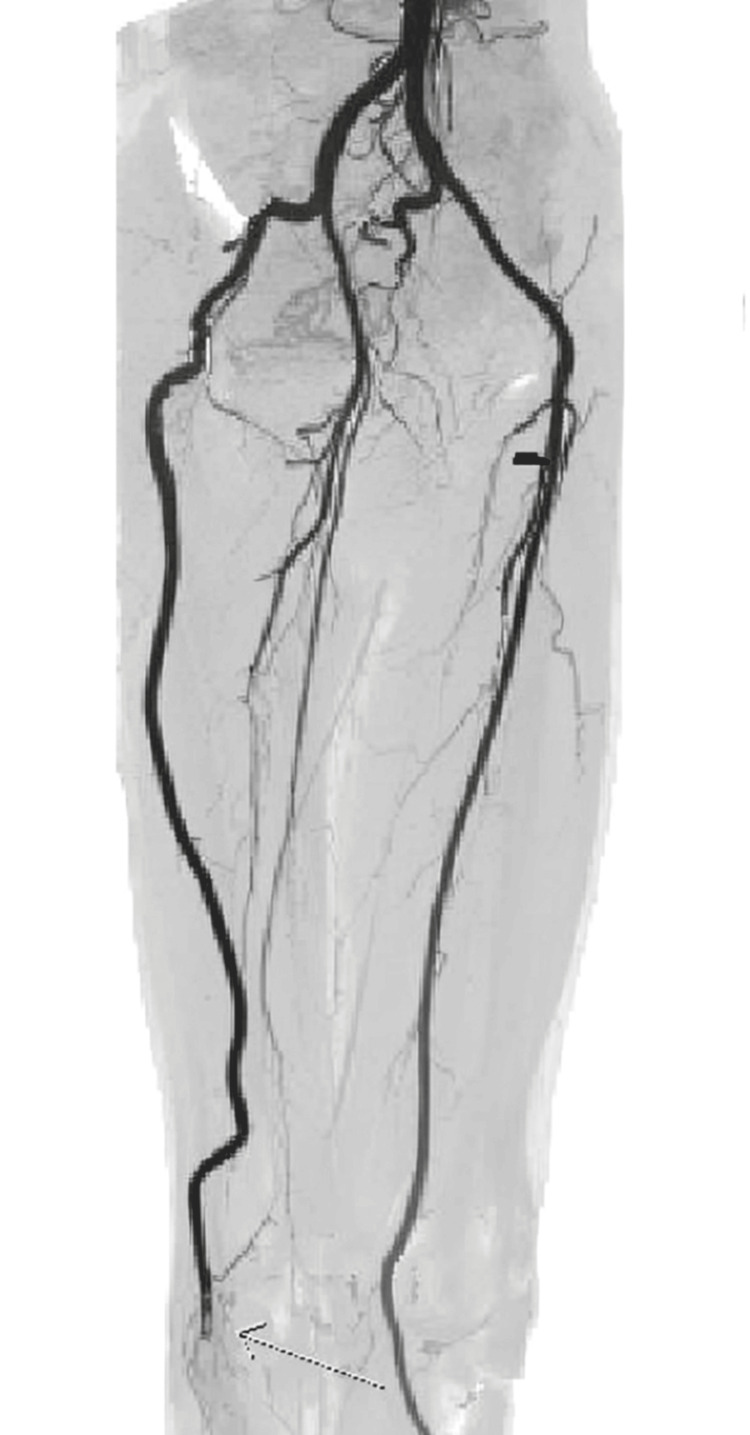
Coronal reformatted pointed (arrows in images) to the level of complete occlusion of PSA.

Notably, a large collateral vessel was identified, arising from the right internal iliac artery. This collateral vessel followed a course behind the thigh before reconnecting with the proximal popliteal artery. Remarkably, the caliber of this collateral vessel matched that of the contralateral normal superficial femoral artery. Based on the diagnostic findings, the patient was diagnosed with a right-sided PSA, an exceedingly rare congenital vascular anomaly where the embryonic sciatic artery persists (Figures 3-5 showing patent lumen and Figures 6-8 showing thrombus and occluded PSA).

**Figure 9:**
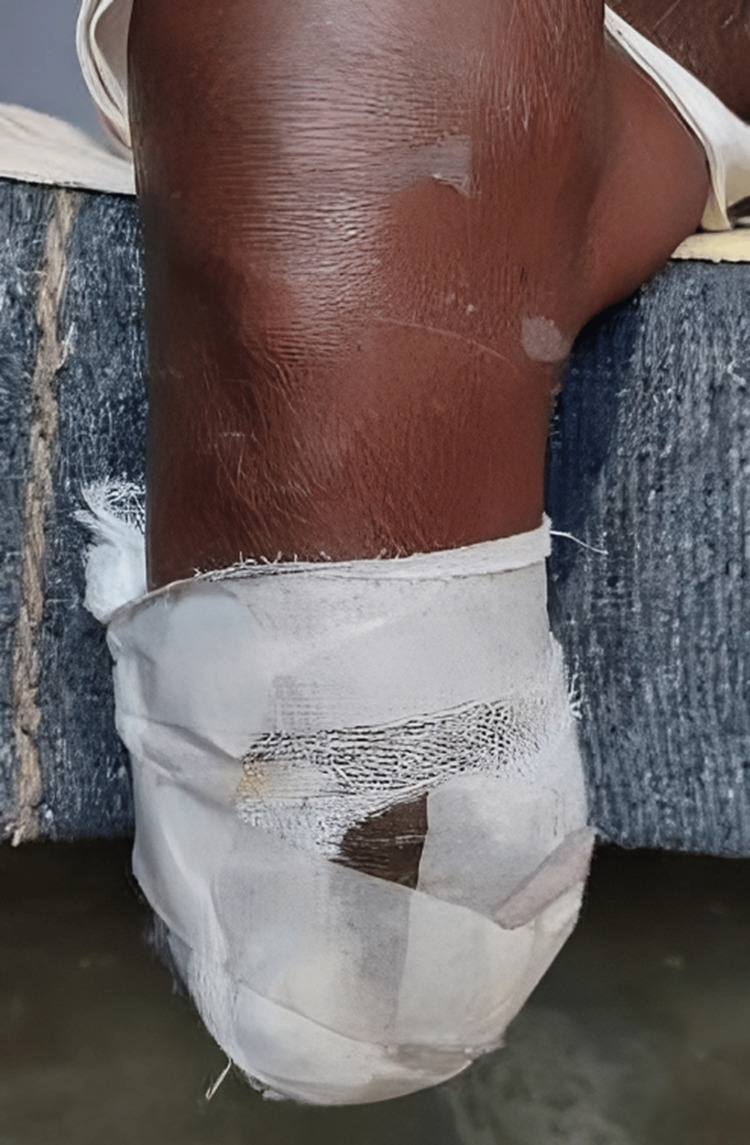
Immediate postop (postoperative) picture showing below-knee amputation.

Given the severity of the irreversibility of the ischemic condition, a below-knee amputation was performed (Figure 9).

**Figure 10:**
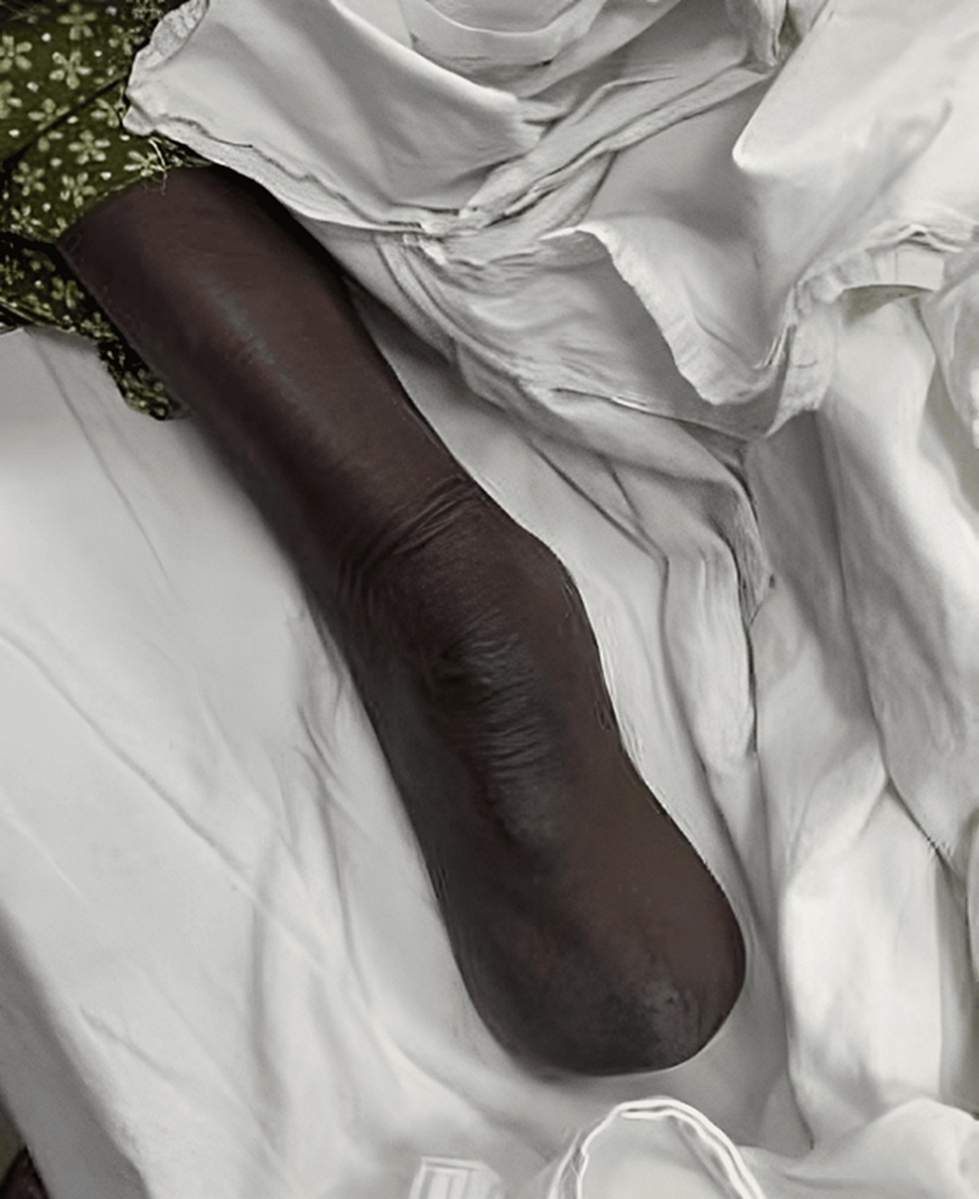
Four months after below-knee amputation.

The patient was followed up for further care and occupational physiotherapy (Figure 10).

## Discussion

The presence of a PSA in adults represents a rare and fascinating vascular anomaly with unique clinical implications. Understanding its embryonic origins, anatomical course, and associated complications is essential for healthcare providers managing patients with this condition.

The sciatic artery is a crucial vascular structure during the embryonic phase, serving as the main axial artery responsible for supplying blood to the lower limbs. Typically, it undergoes regression by the 22-mm stage of embryonic development. At this point, the femoral artery assumes the role of the principal blood supply to the lower limbs as part of the normal developmental process [8].

In cases where the regression of the sciatic artery does not occur as expected, it persists into adulthood as a PSA. The course of the PSA in adults is distinctive and often tortuous. It descends inferiorly beneath the gluteus maximus muscle, passes posteriorly to the greater trochanter of the femur, and courses along the posterior aspect of the adductor magnus muscle until it reaches the popliteal fossa. Here, it assumes the role of the popliteal artery. This atypical course exposes the PSA to various mechanical stresses, including stretching and trauma [9].

PSA is associated with several clinical challenges and potential complications. A significant proportion of individuals with PSA (44-61%) develop aneurysmal changes [10]. These changes can be attributed to the anatomical course of the artery, which predisposes it to stretch during hip flexion and reduced elastin content in the congenital arterial wall. Aneurysms can lead to thrombosis, rupture, and distal embolization, posing a significant clinical risk [10].

Diagnosing PSA can be challenging and often requires a high level of clinical suspicion by physicians. One characteristic clinical sign, known as Cowie's sign, involves the presence of an absent or diminished femoral pulse with a palpable popliteal pulse [11]. Diagnostic confirmation typically involves angiography [10].

PSA is classified into five distinct types, considering its anatomical variations and relationship with the femoral artery [5]. Treatment options for PSA aneurysms depend on the clinical presentation. For asymptomatic cases, regular follow-up with physical examination and duplex scanning is often sufficient. In symptomatic acute settings, such as thrombosis, a thrombectomy is performed, followed by a staged revascularization or exclusion procedure. However, this approach is associated with a 10%-20% amputation rate, highlighting the complexity and potential severity of acute PSA-related complications [11].

## Conclusions

This case presentation highlights the clinical journey of a patient, who presented with lower limb ischemia due to a thrombosis of PSA. It serves as a poignant reminder for healthcare professionals to remain vigilant for rare anatomical anomalies in the realm of lower limb vascular disorders. Prompt recognition and intervention are pivotal for successful patient outcomes, as exemplified in this complex case.
